# Dietary Vegetable Powders Modulate Immune Homeostasis and Intestinal Microbiota in Mice

**DOI:** 10.3390/foods11010027

**Published:** 2021-12-23

**Authors:** Yixin Zou, Haifei Yu, Li Zhang, Zheng Ruan

**Affiliations:** 1 Beijing Advanced Innovation Center for Food Nutrition and Human Health, Beijing Technology and Business University (BTBU), Beijing 100048, China; zyx_990812@163.com; 2 State Key Laboratory of Food Science and Technology, Institute of Nutrition and School of Food Science, Nanchang University, Nanchang 330047, China; ffeiiii@163.com (H.Y.); zhangli@ncu.edu.cn (L.Z.)

**Keywords:** vegetables, freeze-drying, immunity, intestinal flora, polyphenols

## Abstract

As the largest immune organ of the human body, the intestine also plays a vital role in nutrient digestion and absorption. Some vegetables are considered to have improvement effects on the intestine. This experiment explored the effects of freeze-dried asparagus, broccoli and cabbage powder on the intestinal immune homeostasis and microflora of mice. Thirty-two mice were divided into four groups (*n* = 8), including control group (fed normal diet), asparagus group (fed normal diet with 5% asparagus power), broccoli group (fed normal diet with 5% broccoli power) and cabbage group (fed normal diet with 5% cabbage power). The experiment lasted 21 days. The results showed that the serum immunoglobulin concentration (IgA and IgM) and intestinal cytokine content (like IFN-γ and TNF-α) were increased after vegetable powder supplement. The experiment also detected that vegetable powder supplementation changed intestinal flora and their metabolites (short-chain fatty acid), which showed that the abundance of *Lachnospiraceae* and *Bacteroides* were decreased, while the abundance of *Firmicutes* and *Lactobacillus* as well as propionic acid and butyric acid contents were increased. Together, these vegetable powders, especially cabbage, changed the intestinal immune response and microbial activity of mice.

## 1. Introduction

The intestine is the body’s largest immune organ, as well as an important place for digestion and absorption of nutrients. In recent years, intestinal diseases have occurred more and more frequently, and the number of patients with colitis and colorectal adenoma is gradually increasing. In recent investigations of human cancers, colorectal cancer is increasing year by year in global incidence and has occupied the third position [1]. For this reason, maintaining a healthy gut is important. There is a special group of organisms that plays a very key role in the intestinal tract called “the intestinal flora”, which contains about 10 trillion microorganisms [2]. These microorganisms form a symbiotic system with the intestinal tract and participate in a series of physiological processes such as digestion and immunity. Numerous studies have shown that intestinal microbes are closely related to the development of intestinal diseases, and some of them have been found to be involved in the deterioration of colorectal cancer (CRC), such as *Fusobacteriumnuleatum*, *Escherichia coli*, *Bacteroides fragilis*, *campylobacter jejuni*, etc. [3]. Other bacteria can delay the development of CRC by regulating immune response, improving intestinal barrier function and inhibiting cell proliferation. For example, *Lactobacillus acidophilus* and *Bifidobacterium longum* can increase the number of cell conjunctions, and *Lactobacillus casei* can reduce the heteromorphism of patients undergoing colorectal cancer resection [4]. Some bacteria that produce butyric acid can mediate immune system changes through metabolism to balance intestinal cell proliferation and death [5]. Studies over the years have shown that diet can improve the development of intestinal diseases through the intestinal flora. For example, a high-fiber diet can increase the abundance of *Firmicutes* in the intestinal tract, reduce the abundance of *Bacteroidetes* and increase the concentration of short-chain fatty acids, which can promote the effect of immunotherapy; nulin and fructose-oligosaccharides added to the human diet can stimulate the proliferation of bifidobacteria [6]. Inspired by this, we found some vegetables capable of protecting the intestine, such as cruciferous broccoli (*Brassica oleracea var. italica*) and cabbage (*Brassica oleracea*—*Capitata Group*). These kinds of vegetables are rich in vitamins and fiber, and can promote intestinal peristalsis and improve inflammation; they also contain glucosinolates, substances in the body after digestion that can enhance immune function and improve the balance of intestinal flora [7,8]. Asparagus (*Asparagus officinalis*) is also rich in dietary fiber and oligosaccharides; some of its active ingredients such as quercetin and rutin can affect the composition of colon microbes and play a certain role in reducing intestinal inflammation and injury [9]. As these three vegetables can improve the intestinal tract to some extent according to previous reports, we made asparagus, broccoli and cabbage into powder to explore the effects of dietary vegetable powder on the intestinal tract of mice, and to compare the effects of different vegetables. Hopefully our study can provide data support for the development of functional food to improve intestinal health by using these vegetables.

## 2. Materials and Methods

### 2.1. Qualification of Average Daily Body Weight Gain (ADG) of Mice

**Figure 1:**
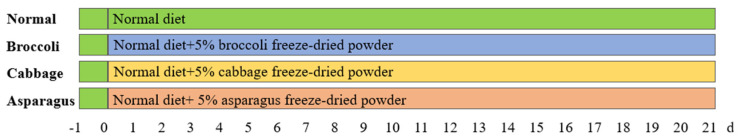
Experimental period diagram.

Animals, diet and experimental design. Procedures for animal experiments are shown in Figure 1. Thirty-two six-week-old KunMing mice were bought from Tian Qin Biotechnology (Changsha, China). All animals were fed in a controlled environment, with temperature 24 ± 1 °C, humidity 40–60%, and 12 h daylight cycle. Then, the mice were randomly divided into 4 groups (*n* = 8): normal control group, asparagus group, broccoli group and cabbage group. The normal control group was intragastric with normal saline and fed an ordinary diet every day, while the other three groups were fed an ordinary diet with 5% freeze-dried vegetable powder. The whole feeding process lasted for 21 days. The experiment was approved by the Animal Experimentation Ethical Committee of Nanchang University (permission number: 201800022). The feeding and operation of experimental animals were conducted in accordance with the experimental animal welfare ethical code of Nanchang University.

After 21 days of feeding, all mice fasted overnight. All mice were euthanized by carbon dioxide anesthesia followed by cervical dislocation, then we removed their intestinal tissue and collected blood and feces into separate EP tubes and stored in a −80 °C refrigerator.

Weight measurement of mice. We weighed the mice in each group at a fixed time every day, and then calculated the average daily gain of mice.

### 2.2. Determination of Immunoglobulin Concentration

The concentrations of immunoglobulin A, G and M in mice serum were determined by double-antibody sandwich enzyme-linked immunosorbent assay (ELISA) referring to the method of Lindsey et al. with some modifications [10].

Serum sample collection. The blood of mice in each group was collected in the corresponding test tube, placed at room temperature for 2 h, centrifuged at 3000 rpm for 10 min, and the supernatant was taken to obtain serum samples.

ELISA determination. ELISA kits (Jiangsu Meimian Industrial Co., Ltd., Zhangjiagang, China) corresponding to IgA, IgG and IgM were taken out to prepare standard solution with concentration gradient for subsequent production of standard curves. We added the diluted standard solution from low to high concentration to the 96-well plate, then added the mice serum diluted with sample dilution to the next well; the amount of solution in each well was 100 μL. At the same time, we made two duplicate wells in the plate. We sealed the film plate slowly and shook the liquid evenly in the hole. After incubation for 2 h at 37 °C, the liquid in the well plate was removed, and then washed 2~3 times with washing solution; we carefully dried the liquid with filter paper. We added 100 μL antibody with a pipette gun, incubated for 60 min, added 100 μL chromogenic substrate in a dark room, added the stop solution immediately after the appropriate color, observed the depth of color in the reaction hole, measured the OD value at 450 nm with a microplate analyzer and calculated the cytokine content in the serum of each group of mice.

### 2.3. Determination of Intestinal Cytokine Concentration in Mice

The concentrations of IL-1, IL-10, TNF-α and INF-γ in the jejunum and ileum of mice were also determined by ELISA as previously described [11].

Collection of intestinal tissue samples. Jejunum and ileum tissues of mice in each group were taken (200 mg) and ground in normal saline to prepare tissue homogenates. After centrifugation at 4 °C and 3000 rpm for 10 min, supernatant was taken and put into plastic tubes.

ELISA determination. The ELISA kits (Jiangsu Meimian Industrial Co., Ltd., Zhangjiagang, China) corresponding to IL-1, IL-10, TNF-α and INF-γ were removed, and the same procedure was performed for each kit using the corresponding liquid. A pipetting gun was used to prepare a standard solution with a concentration gradient, and then a standard curve was produced.

We added the homogenate of intestinal tissue diluted with sample diluent to a 96-well plate at 100 μL per well, then added 100 μL corresponding secondary antibody to the well, shaking to make the liquid uniform. After incubation at 37 °C for 1 h, the washing solution was washed 2–3 times, adding 80 μL affinity enzyme-HRP, incubating for 30 min, adding 50 μL color substrate A and B for color, adding the stop solution quickly after the appropriate color, observing the depth of color in the reaction hole and measuring the OD value at 450 nm with a microplate meter. The contents of cytokines in jejunum and ileum homogenates were calculated.

### 2.4. Quantification of Short-Chain Fatty Acids (SCFAs)

The concentrations of acetic acid, propionic acid and butyric acid in caecum were determined by gas chromatography as per Wang et al. [12].

Short-chain fatty acids were extracted from cecal chyme with 0.05% phosphoric acid [13], mixed evenly and shaken, and stood at room temperature for 20 min. After centrifuging at 15,000 rpm at 4 °C for 15 min, the supernatant was passed through gas chromatography and a flame ionization detector. At the same time, the curve of acetic acid, propionic acid and butyric acid standard solution was made, and then the sample results were compared with the standard results to determine the content of each short-chain fatty acid.

### 2.5. Determination of Intestinal Flora in Mice

Referring to the determination method of Yang et al. [14], we used a high-throughput 16S rRNA gene sequencing method to measure the intestinal flora of mice. The datasets generated for this study can be found in the National Center of Biotechnology Information (NCBI) Sequence Read Archive (SRA) database (NCBI BioProject PRJNA773049).

DNA extraction. To accurately weigh 200 mg feces, we used a QIAamp DNA Stool Mini Kit (Qiagen, Hilden, Germany) and extracted the DNA according to kit instructions.

MiSeq Illumina Sequencing. Primers 515F (5′-GTGCCAGCMGCCGCGGTAA-3′) and 926R (5′-GGATACHVGGGTWTCTAAT) were used to determine the sequencing range in the V3 and V4 regions of rRNA. We then used a MiSeq PE250 instrument (Illumina, San Diego, CA, USA) to sequence the purified amplicon. After that, sequences were assigned to operational taxonomic units (OTUs) by UPARES at 97% similarity. The results were then analyzed for species classification and abundance, and the phylum level heat map of species composition, beta diversity analysis and two-dimensional plan of PCoA were drawn. At the same time, the differences in the number of microorganisms among groups were compared and a Venn diagram was drawn.

### 2.6. Statistics Analysis

The data obtained in this study were analyzed by SPSS statistical software 24.0. To compare the mean differences among the groups, one-way analysis of variance (ANOVA) was used. Differences were considered significant at *P* < 0.05. All values are expressed as the mean ± SD.

## 3. Results

### 3.1. Calculation of the Average Daily Gain of Mice in Each Group

The statistical average daily gain of mice in each group is detailed in Table 1. It can be seen that the average daily gain of mice in the normal control group was the largest, which was 0.855 g. The daily weight gain of mice in the other three groups after feeding with freeze-dried vegetable powder decreased, and the descending order was cabbage group > asparagus group > broccoli group; the average daily weight gain of mice in the cabbage group was only 0.737 g. However, statistical analysis showed that there was no significant difference in weight loss among all groups.

### 3.2. Analysis of Immunoglobulin Concentration in Mice

Table 2 shows the measured changes of immunoglobulin A, G and M in each group of mice. It is not difficult to see that compared with the control group, dietary vegetable powder supplementation changed the immunoglobulin content in the intestinal tract of mice, and the IgM and IgA concentrations of the three groups were increased. The average IgA concentration of the asparagus (88.05 ng/mL) and cabbage (84.86 ng/mL) feeding groups as well as the lgM concentration of the asparagus (4.67 ng/mL) and broccoli (4.63 ng/mL) feeding groups were significantly higher than the control group (80.12 ng/mL and 4.28 ng/mL, respectively); the IgA concentration of the broccoli feeding group increased to 93.21 ng/mL, higher than the other three groups. Although the lgM concentration of the cabbage feeding group increased, the change was less obvious than that of the other two vegetable feeding groups. The concentrations of lgG in the four groups were not significantly different.

### 3.3. Analysis of Cytokine Concentration in Jejunum and Ileum of Mice

To investigate the effect of freeze-dried vegetable powder on the intestinal tract of mice, we measured the concentration of cytokines in the jejunum and ileum of mice in each group. According to Table 3, in the jejunum the concentrations of IL-1 (80.27 pg/mL) and TNF-α (950.23 pg/mL) were significantly increased and the concentrations of IL-10 (1326.68 pg/mL) were obviously decreased after asparagus feeding. At the same time, the concentration of IL-1 (61.07 pg/mL) decreased significantly after broccoli feeding. Compared with the control group, the concentration of IFN-γ in mice fed with three vegetable powders increased significantly, and the concentration of IFN-γ in the broccoli feeding group increased the most (1131.92 pg/mL). In the ileum, the concentrations of IFN-γ and IL-10 were significantly increased by asparagus feeding (1066.44 pg/mL and 1338.77 pg/mL, respectively), and the concentration of TNF-α was significantly increased by broccoli feeding (928.09 pg/mL). Meanwhile, IL-1 and TNF-α concentrations (100.39 pg/mL and 829.00 pg/mL, respectively) were significantly increased in the cabbage feeding group.

### 3.4. Analysis of Short-Chain Fatty Acid Content in Cecum of Mice

In order to study whether the intake of vegetable powder affected the intestinal flora of mice, the content of short-chain fatty acids in the intestinal tract of mice was also measured in this experiment, as shown in Table 4. Compared with the control group (6.03 μmol/g), the content of acetic acid in the cecum of asparagus and broccoli was slightly decreased (4.67 μmol/g and 4.87 μmol/g respectively), and that of the cabbage feeding group was 6.40 μmol/g. Although it was higher than that of the other two groups, there was no statistical difference in caecal acetic acid content in mice fed with the three vegetable powders compared with the control group. After cabbage feeding, the propionic acid content in mice (3.59 μmol/g) was obviously increased compared with other groups, and the butyric acid content in mice after the cabbage feeding group (2.82 μmol/g) was significantly increased compared with other groups.

### 3.5. Analysis of High-Throughput 16S rRNA Gene Sequencing Results

**Figure 2:**
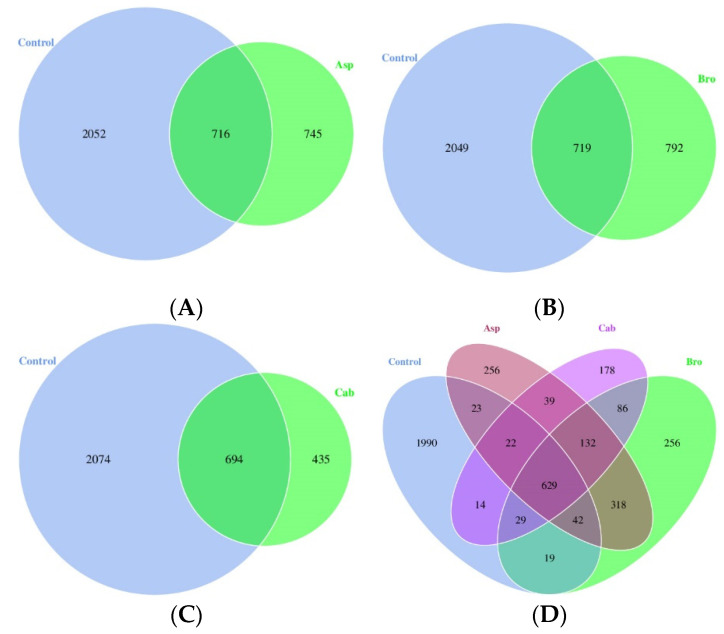
Venn diagram of intestinal flora species in mice. (**A**) Comparison of microflora abundance between control group and cabbage group; (**B**) comparison of microflora abundance between control group and broccoli group; (**C**) comparison of microflora abundance between control group and asparagus group; (**D**) comparison of microflora abundance between four groups.

To compare whether the composition of intestinal flora caused differences among groups, the species distribution of intestinal flora of mice in each group was drawn into a Venn diagram, as shown in Figure 2A–D. As can be seen from Figure 2A–C, the types of intestinal flora of mice in the blank control group were more than those in the other three groups, with an average of more than 2000 kinds. In the three groups treated with vegetable powder, the types of intestinal flora not only decreased to only 1100–1500 kinds, but also changed after the mice were fed with vegetable powder. Only about 700 species of bacteria in each group were the same as those in the blank control group, but new species of bacteria that were not previously present in the blank control group appeared, especially after broccoli feeding; 792 new species of bacteria were detected that were different from the control group. Compared with the control group, as shown in Figure 2D, 256 species of new bacteria emerged after feeding with asparagus, which were different from the other three groups. Similarly, 256 and 178 species of new bacteria emerged after feeding with broccoli and cabbage, respectively, and the quantity of the same species of new bacteria that emerged after feeding with three groups of vegetable powder was 132.

**Figure 3:**
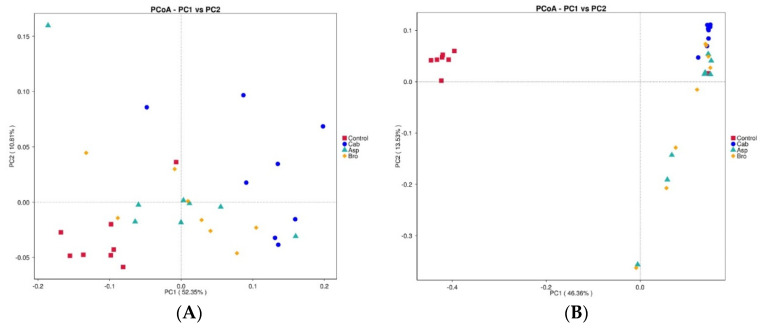
Correlation analysis diagram of intestinal flora species in each group of mice. (**A**) PCoA diagram of a certain angle; (**B**) PCoA diagram of another angle.

The results of beta diversity analysis are shown in Figure 3A,B. As can be seen from the two figures, the bacterial abundance of the blank control group was significantly different from that of the other three groups. The bacterial abundance of the other three groups after being fed with vegetable powder had a certain correlation, among which the bacterial abundance of the mice after being fed with asparagus and broccoli had a greater correlation, while the bacterial abundance of the cabbage group was slightly different.

**Figure 4:**
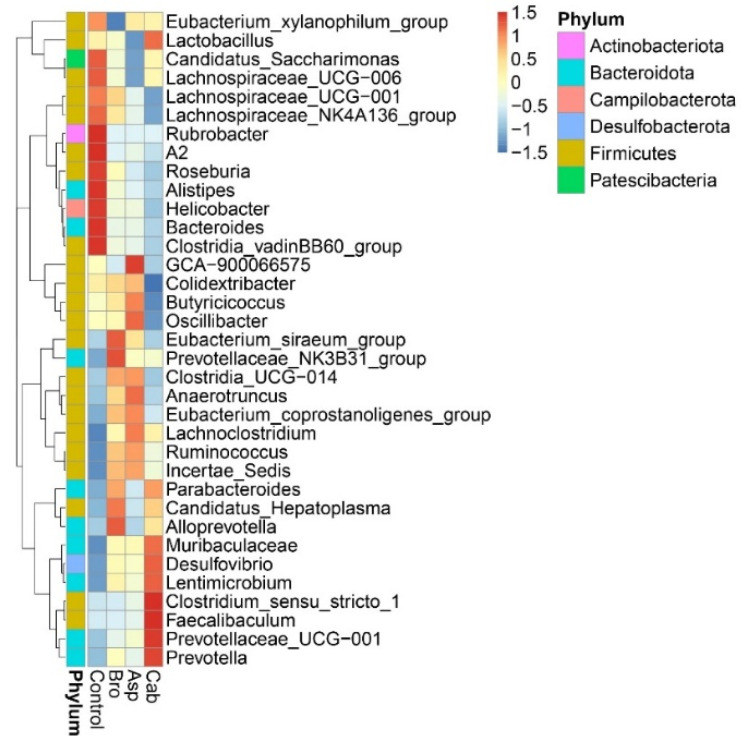
Heat map of intestinal flora abundance in mice.

The thermal map of the horizontal composition of the microphyla in mice was further drawn, as shown in Figure 4. The differences in the composition of the microphyla in each group can be intuitively seen. As can be seen from the figure, the intestinal flora of mice are concentrated in the following six phyla: *Actinobacteriota*, *Bacteroidota*, *Campilobacterota*, *Desulfobacterota*, *Firmicutes* and *Patescibacteria*, and mostly *Firmicutes*, *Bacteroidota* followed. In the blank control group, bacteria genera with a high abundance in mice included *Lachnospiraceae*, *Rubrobacter*, *Roseburia*, *Alistipes*, *Helicobacter*, *Bacteroides*, *Clostridia*, etc. The genera with low abundance were *Lachnoclostridium*, *Ruminococcus*, *Muribaculaceae* and *Desulfovibrio*. In the heat map, the composition and abundance of the bacteria community after feeding with vegetable powder were significantly different from that of the control group. The abundances of bacteria with a higher abundance in the control group were almost all decreased in the three groups. For example, the abundances of several bacteria with higher abundances in the control group just mentioned were significantly decreased in the cabbage treatment group. In contrast, the low abundance of bacteria in the control group seemed to rebound after being fed with vegetable powder; for example, in the cabbage group, the abundance of *Muribaculaceae*, *Desulfovibrio*, *Clostridium*, *Faecalibaculum*, *Prevotellaceae*, *Prevotella* and *Lactobacillus* increased significantly. After broccoli treatment, the abundance of *Eubacterium* and *Alloprevotella* increased, while the abundance of *Oscillibacter*, *Anaerotruncus* and *Lachnoclostridium* increased obviously in the asparagus feeding group.

## 4. Discussion

### 4.1. Dietary Intake of Vegetable Powder Increased Immunoglobulin Concentration in Mice

Brassica vegetables (cabbage and broccoli) have been reported to be a good source of many nutrients and phytochemicals that are effective in preventing the development of certain immune dysfunctional diseases [15]. In addition, Zhang et al. [16] showed that *Lactobacillus plantarum* NCU116 fermentation on *Asparagus officinalis* polysaccharide also had good immunomodulatory activity. Notably, immunoglobulins are the most widely used blood products in clinical practice due to their unique immunomodulatory and anti-inflammatory activities [17]. Liu et al. [18] found that broccoli residues fermented with probiotics increased the levels of Ig A, Ig G and Ig M in the sera of *C. perfringens*-infected birds. Similarly, our results showed that broccoli, cabbage and asparagus powder all increased Ig A and Ig M levels in mice serum, but did not have a significant effect on Ig G levels (Table 2). In contrast to our experimental results, Miyazaki et al. [19] found that 1% fermented extract of cabbage was able to increase significantly the level of Ig G in the serum of rats. This may be due to the fact that fermentation alters the nutrient content of cabbage and thus its effect on immune function. One of the important components of the intestinal barrier function of the intestinal flora structure is the host secretory immune system, which limits opportunistic invasion by pathogenic members of the intestinal flora [20]. Ig A is specifically used for the protection of intestinal mucosa and is the most abundant antibody isotype in mucosal secretions [21]. Many reports have shown that prebiotics can maintain the homeostasis of the immune environment and improve intestinal barrier function by affecting the production and secretion of Ig A [22,23,24]. Importantly, our study found that broccoli, cabbage and asparagus powder were most effective in increasing serum Ig A levels compared to serum Ig G and Ig M levels. This suggests that dietary vegetable powders are promising candidates for prebiotics, which in turn regulate human intestinal immune homeostasis.

### 4.2. Dietary Intake of Vegetable Powder Changed the Concentration of Cytokines in the Jejunum and Ileum of Mice

Cytokines, including tumor necrosis factor (TNF), interleukins (ILs) and interferons (IFN), can affect the body by modulating the immune system, thereby regulating cell proliferation and repairing damaged tissue [25]. To further investigate the modulation of intestinal immune activity by vegetable powder, we measured the cytokines (IL-1, IL-10, TNF-α, and INF-γ) secreted from the jejunum and the ileum of mice after consuming vegetable powder. In the present study, we found that the concentration of IL-1 was higher in the ileum in the cabbage group than that in the control group. This suggests that cabbage powder regulates the immune response in the ileum, which in turn is involved in the maintenance of immune homeostasis in the intestine [26]. IL-10 is an important immune regulator of many autoimmune diseases and inflammatory conditions [7]. Our results show that IL-10 concentrations in the jejunum of mice fed broccoli, cabbage and asparagus powder were significantly lower compared to controls. However, Hubbard et al. [9] suggested that dietary broccoli could reduce colitis and maintain intestinal environmental homeostasis in mice because broccoli protects the intestine by regulating aryl hydrocarbon receptor (AHR) activity. In addition, Power et al. [27] showed that dietary cooked asparagus was effective in reducing colonic inflammation and repairing colonic mucosal damage. In conclusion, vegetable powder has a moderating effect on maintaining the homeostasis of the intestinal environment. However, the mechanism of the interaction between the anti-inflammatory effect of vegetable powder on the intestinal tract and the immune response needs to be further investigated. IFN-γ has anti-infective and anti-proliferative properties and is also a key regulator of immune cell activation [28,29]. Currently, some plant polysaccharides have been reported to demonstrate the superior ability to promote IFN-γ secretion [30,31]. In addition, the results of this study demonstrate that broccoli, cabbage and asparagus powder all promoted the secretion of IFN-γ in the jejunum of mice. This suggests that dietary vegetable powders can promote immunity by modulating inflammatory cytokines in the jejunum and ileum.

### 4.3. Dietary Intake of Vegetable Powder Changed the Composition and Abundance of Intestinal Flora in Mice

The balance of intestinal flora structure is considered to be an important factor affecting host health, and diet is the main determinant of intestinal flora composition. Dietary intake of fruits and vegetables has been reported to influence the composition of intestinal microorganisms [32]. In particular, it has been shown that cruciferous vegetables, like cabbage and broccoli, alter the composition of the human gut flora when consumed [33]. This study found that, compared to mice fed a normal diet, dietary cabbage powder increased the intestinal abundance of *Muribaculaceae*, *Desulfovibrio*, *Clostridium*, *Faecalibaculum*, *Prevotellaceae*, *Prevotella* and *Lactobacillus* abundance in the intestine of mice (Figure 4). The genera *Muribaculaceae*, *Clostridium*, *Faecalibaculum* and *Prevotellaceae* are all thought to be involved in the synthesis of SCFAs and to have a protective effect on the intestinal mucosal barrier [34,35,36]. Particularly, *Clostridium* is considered to be one of the important families of butyric acid producing bacteria [37], which is consistent with the above results. *Prevotella* is a bacterium that benefits the human intestine by promoting the release of inflammatory mediators from immune cells and various stromal cells [38,39]. *Lactobacillus* is known to be a probiotic that improves intestinal inflammation and protects the intestinal barrier [40,41]. In addition, Mu et al. [42] suggested that *Lactobacillus* was one of the reasons for the decrease in intestinal microflora in mice. As shown in Figure 2, the species of intestinal flora were significantly reduced in the cabbage group compared to the control group. Therefore, the decrease in intestinal flora species in mice fed cabbage powder may be associated with an increase in the abundance of *Lactobacillus*.

Broccoli and asparagus powder increased the abundance of *Alloprevotella*, *Eubacterium*, *Oscillibacter*, *Anaerotruncus* and *Lachnoclostridium* spp. in the intestine (Figure 4). Among them, *Oscillibacter* was able to promote the production of anti-inflammatory metabolites [43]. In addition, *Alloprevotella* is an efficient degrading bacterium of dietary fiber, which metabolizes dietary plant-derived polysaccharides into SCFAs [44]. *Lachnoclostridium*, *Eubacterium* and *Anaerotruncus* spp. can all be used as SCFA-producing bacteria, and both *Eubacterium* and *Anaerotruncus* spp. have been shown to be butyrate-producing bacteria in the human intestine [45,46,47]. This flora-regulating effect indicates that the vegetable powder has prebiotic properties.

Admittedly, there are some limitations in this study that could be addressed in future research. First, only several cytokines and immunoglobulin were selected for determination in the experimental process because of the limitations of experimental design. This experiment was only to preliminarily explore the effects of freeze-dried powder of asparagus, broccoli and cabbage on the intestinal tract of mice. Although the measurement indexes were not comprehensive enough, the selected measurement indexes in the experiment were representative to some extent. Compared with the control group, the significant changes in these indexes could reflect that the intake of vegetable powder changed the intestinal immune environment of mice, which was also in line with our preliminary assumption. Finally, the influence and pathway of the active ingredients of vegetable powder on intestinal microbiota and its metabolites, and the effect and molecular mechanism of the active ingredients or microbiota-driven metabolites on the intestinal cells of the host need further research. Based on the results of this experiment, we need to take these limitations into account in the follow-up work and design a more perfect experimental scheme for further studying the specific action mechanism of vegetable powder on the intestinal tract of mice.

## 5. Conclusions

In conclusion, asparagus, broccoli and cabbage powder altered propionic and butyric acid contents, immunoglobulin concentrations (IgA and IgM), cytokine concentrations (IL-1, TNF-α, IFN-γ, IL-10) and microbial composition in mice. These results indicate that dietary vegetable powder may affect the immune homeostasis in the intestinal tract of mice and change the intestinal microflora of mice, which provides certain data support for the subsequent development of functional food or nutritional food rich in vegetable active ingredients.

## Figures and Tables

**Table 1:** The average daily body weight gain (ADG) of mice in different groups.

Group	ADG (g/day)
Control	0.855 ± 0.036
Asparagus	0.816 ± 0.039
Broccoli	0.788 ± 0.025
Cabbage	0.737 ± 0.043

Values are means with standard deviations (*n* = 8).

**Table 2:** Effects of vegetable powder on serum immunoglobulin concentrations of mice.

Group	Control	Asparagus	Broccoli	Cabbage
IgA, ng/mL	80.12 ± 0.82 ^c^	88.05 ± 1.21 ^b^	93.21± 1.05 ^a^	84.86 ± 0.93 ^b^
IgG, ng/mL	813.45 ± 9.83	838.03 ± 12.02	823.11 ± 9.84	827.20 ± 11.82
IgM, ng/mL	8.56 ± 0.15 ^b^	9.34 ± 0.14 ^a^	9.26 ± 0.08 ^a^	8.87 ± 0.13 ^a^^b^

Values are means with standard deviations (*n* = 8). Means in the same row with different superscripts differ (*P* < 0.05).

**Table 3:** Effects of vegetable powder on intestinal cytokine concentrations of mice.

Group	Control	Asparagus	Broccoli	Cabbage
Jejunum, pg/mL				
IL-1	69.02 ± 1.17 ^b^	80.27 ± 1.47 ^a^	61.07 ± 1.61 ^c^	68.84 ± 1.23 ^b^
IL-10	1676.67 ± 36.71 ^a^	1326.68 ± 17.17 ^c^	1494.44 ± 23.72 ^b^	1589.64 ± 23.35 ^ab^
TNF-α	728.13 ± 14.92 ^b^	950.23 ± 18.45 ^a^	729.37 ± 17.83 ^b^	918.64 ± 8.94 ^a^
IFN-γ	762.96 ± 23.15 ^c^	889.94 ± 11.79 ^b^	1131.92 ± 18.49 ^a^	1071.57 ± 10.54 ^a^
Ileum, pg/mL				
IL-1	65.39 ± 2.71 ^b^	70.62 ± 1.47 ^b^	68.37 ± 1.69 ^b^	100.39 ± 1.41 ^a^
IL-10	1629.44 ± 44.49 ^a^	1338.77 ± 19.50 ^b^	1546.33 ± 20.21 ^a^	1228.75 ± 24.14 ^b^
TNF-α	713.17 ± 13.56 ^c^	762.04 ± 14.36 ^c^	928.09 ± 16.00 ^a^	829.00 ± 13.79 ^b^
IFN-γ	735.64 ± 6.64 ^b^	1066.44 ± 19.37 ^a^	792.01 ± 19.42 ^b^	765.54 ± 16.22 ^b^

Means in the same row with different superscripts differ (*P* < 0.05).

**Table 4:** Effects of vegetable powder on intestinal short-chain fatty acid concentrations of mice.

Group	Control	Asparagus	Broccoli	Cabbage
Caecum, μmol/g				
Acetic acid	6.03 ± 0.36 ^ab^	4.67 ± 0.23 ^b^	4.87 ± 0.32 ^b^	6.40 ± 0.47 ^a^
Propionic acid	1.70 ± 0.20 ^b^	2.00 ± 0.14 ^b^	2.02 ± 0.18 ^b^	3.59 ± 0.33 ^a^
Butyric acid	1.48 ± 0.22 ^b^	1.95 ± 0.18 ^ab^	2.16 ± 0.38 ^ab^	2.82 ± 0.38 ^a^

Means in the same row with different superscripts differ (*P* < 0.05).

## Data Availability

The datasets generated for this study can be found in the National Center of Biotechnology Information (NCBI) Sequence Read Archive (SRA) database (NCBI BioProject PRJNA773049).
